# Prediction of Microvascular Invasion and Its M2 Classification in Hepatocellular Carcinoma Based on Nomogram Analyses

**DOI:** 10.3389/fonc.2021.774800

**Published:** 2022-01-14

**Authors:** Shengsen Chen, Chao Wang, Yuwei Gu, Rongwei Ruan, Jiangping Yu, Shi Wang

**Affiliations:** ^1^ Department of Endoscopy, Cancer Hospital of the University of Chinese Academy of Sciences (Zhejiang Cancer Hospital), Institute of Cancer and Basic Medicine (IBMC), Chinese Academy of Sciences, Hangzhou, China; ^2^ Department of Emergency, Huashan Hospital affiliated to Fudan University, Shanghai, China; ^3^ Department of Rehabilitation Medicine, Huashan Hospital affiliated to Fudan University, Shanghai, China

**Keywords:** hepatocellular carcinoma, microvascular invasion (MVI), M2 classification, prediction model, nomogram

## Abstract

**Background and Aims:**

As a key pathological factor, microvascular invasion (MVI), especially its M2 grade, greatly affects the prognosis of liver cancer patients. Accurate preoperative prediction of MVI and its M2 classification can help clinicians to make the best treatment decision. Therefore, we aimed to establish effective nomograms to predict MVI and its M2 grade.

**Methods:**

A total of 111 patients who underwent radical resection of hepatocellular carcinoma (HCC) from January 2017 to December 2019 were retrospectively collected. We utilized logistic regression and least absolute shrinkage and selection operator (LASSO) regression to identify the independent predictive factors of MVI and its M2 classification. Integrated discrimination improvement (IDI) and net reclassification improvement (NRI) were calculated to select the potential predictive factors from the results of LASSO and logistic regression. Nomograms for predicting MVI and its M2 grade were then developed by incorporating these factors. Area under the curve (AUC), calibration curve, and decision curve analysis (DCA) were respectively used to evaluate the efficacy, accuracy, and clinical utility of the nomograms.

**Results:**

Combined with the results of LASSO regression, logistic regression, and IDI and NRI analyses, we founded that clinical tumor-node-metastasis (TNM) stage, tumor size, Edmondson–Steiner classification, α-fetoprotein (AFP), tumor capsule, tumor margin, and tumor number were independent risk factors for MVI. Among the MVI-positive patients, only clinical TNM stage, tumor capsule, tumor margin, and tumor number were highly correlated with M2 grade. The nomograms established by incorporating the above variables had a good performance in predicting MVI (AUC_MVI_ = 0.926) and its M2 classification (AUC_M2_ = 0.803). The calibration curve confirmed that predictions and actual observations were in good agreement. Significant clinical utility of our nomograms was demonstrated by DCA.

**Conclusions:**

The nomograms of this study make it possible to do individualized predictions of MVI and its M2 classification, which may help us select an appropriate treatment plan.

## Introduction

Primary liver cancer is one of the most common cancers worldwide and globally ranks fifth and fourth in morbidity and mortality, respectively (1). In China, liver cancer was reported as the fourth most common cancer in 2015, and its mortality ranked second among malignant tumors (2), with approximately 466,100 new cases and 422,000 deaths (3). As the most common type of liver cancer, hepatocellular carcinoma (HCC) has high invasiveness, and its 5-year recurrence rate after surgery is nearly 70% (4, 5), which results in a poor prognosis (6). Despite the diagnosis and treatment of HCC having been greatly improved, recurrence within 5 years after operation still remains a huge challenge (7). Microvascular invasion (MVI), an indicator (only diagnosed by histopathological examination) of HCC aggressive behavior (8), is defined as the cancer cell nest appearing in vessels lined with endothelium under microscopy (9, 10). When MVI is present, tumor cells can spread and metastasize in the liver, forming portal vein tumor thrombi or multiple lesions or distant metastasis (11). So MVI is considered as a critical pathological factor correlated with tumor recurrence and survival (12) and has been used as a prognostic reference index in the treatment options for both primary and recurrent HCC (13, 14). In resected HCC specimens, MVI was detected in approximately 7.8% to 74.4% of cases (15), and the MVI detection rate in early HCC varied greatly from 12.4% to 37.3% (16).

Recently, the three-tiered MVI grading system (MVI-TTG) has been proposed and it classifies the specimens as M0 (no MVI), M1 (1–5 sites of MVI and located at ≤1 cm away from the tumor-adjacent liver tissue), and M2 (>5 MVI sites or at >1 cm away from the tumor-adjacent liver tissue) (17). The MVI-TTG scheme is simple and clear, is easy to implement, and can stratify HCC patients in different risks for recurrence and survival (18). In the presence of MVI, HCC patients with M2 classification showed a worse prognosis after radical resection than those with M1 classification. Moreover, the M2 grade of MVI is a high-risk factor for postoperative residual cancer recurrence and intrahepatic metastasis (18). Therefore, we should pay attention not only to the presence or absence of MVI but also to its M2 classification.

If HCC patients who require liver resection are at high risk of MVI, it is recommended to widen the surgical margin to eradicate MVI and improve clinical prognosis (19). When MVI is present and classified as M2 grade, more intense comprehensive treatment such as adjuvant transarterial chemoembolization (TACE) may need to be taken to prevent HCC postoperative recurrence and metastasis (20). Given that MVI, especially its M2 grade, is the poor prognostic factor of HCC (18), there is an urgent need to build effective and accurate prediction models that can predict MVI and its M2 classification to optimize the management of patients (21). A few studies have built and validated some nomograms for MVI prediction, but the inclusion criteria of HCC patients were heterogeneous and so were the clinical characteristics of selected patients in these studies (22). Additionally, the study on the risk prediction of M2 classification in the presence of MVI is still rare currently.

Therefore, in this study, we aimed to determine the effective predictors of MVI and its M2 classification and use these factors to establish corresponding nomograms, which could aid clinicians to select appropriate therapeutic strategies for MVI-positive HCC patients and make the follow-up after curative treatment more targeted.

## Methods

### Patients and Study Design

**Figure 1:**
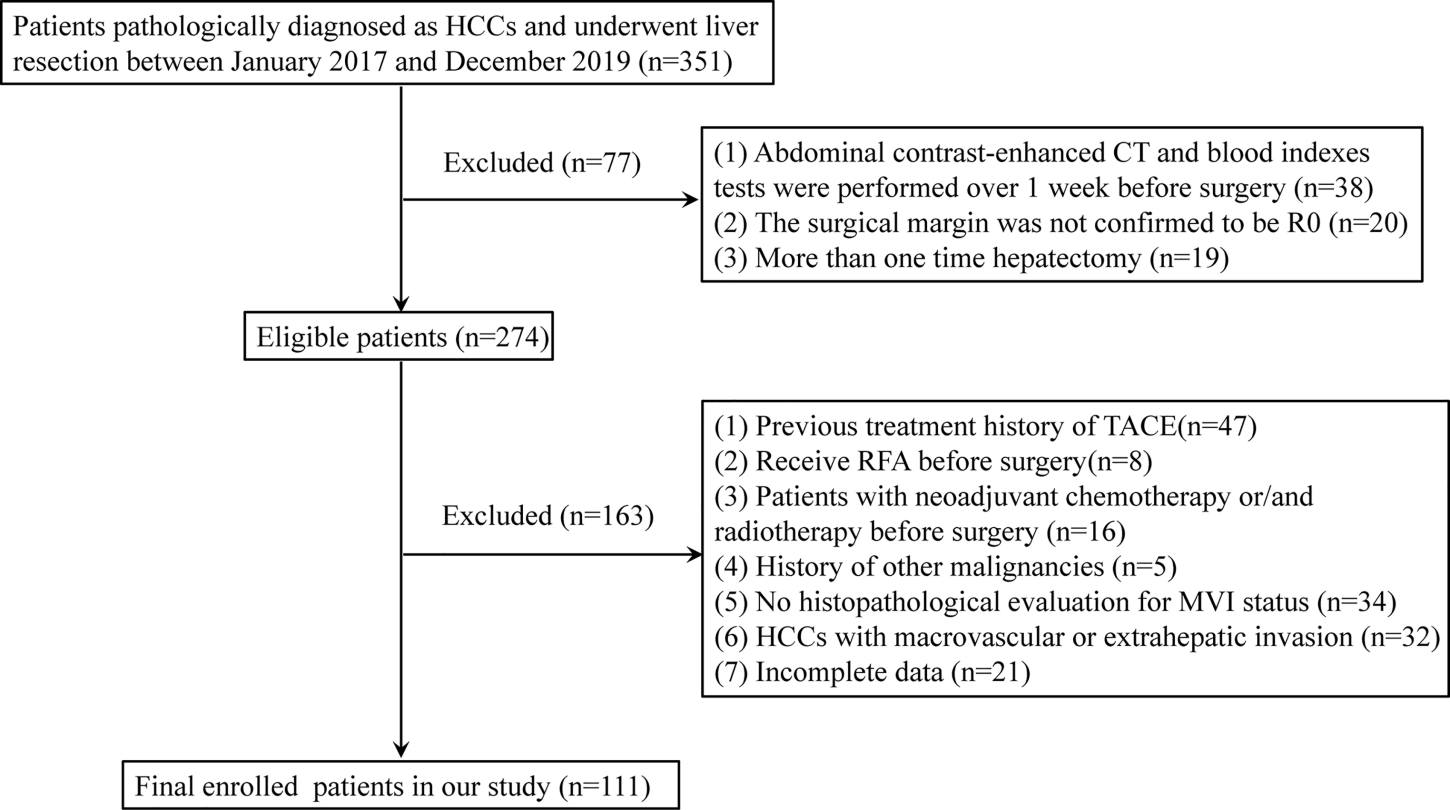
Flowchart of the patients included in the analysis.

We retrospectively collected a total of 111 HCC patients with liver resection from January 2017 to December 2019. The criteria for the exclusion of patients were as follows: 1) abdominal contrast-enhanced CT and blood index tests were performed more than 1 week before surgery; 2) the surgical margin was not confirmed to be R0 defined in a previous report (23); 3) patients underwent hepatectomy more than one time; 4) patients received radiofrequency ablation (RFA), TACE, neoadjuvant chemotherapy, and/or radiotherapy before surgery; 5) patients who have a history of other malignant tumors; 6) MVI status was not evaluated by histopathological examination; 7) HCCs with macrovascular or extrahepatic invasion; and 8) incomplete clinical data. The flowchart of the patient selection is summarized in **Figure 1**.

### Clinical Variables and Pathological Characteristics

Basic information on admission such as age, sex, symptoms at diagnosis, and some laboratory indicators was collected including blood routine test, liver and kidney function, hepatitis B tests, and tumor markers. Besides, data of tumor size, liver cirrhosis, number of HCC lesions, tumor location, tumor margin, and tumor capsule were extracted from the results of preoperative abdominal contrast-enhanced CT scans. The cardiopulmonary function was also evaluated by cardiac ultrasound and pulmonary function test to make sure the patients can tolerate the operation. The postoperative tissue specimens were further assessed by pathological examination to confirm the presence or absence of MVI. As described above, patients with positive MVI were classified into M1 and M2 according to the three-tiered MVI grading system (17). Other pathological characteristics like satellite nodule and the Edmondson–Steiner classification were also collected.

### Statistical Analysis

Continuous variables which were expressed as median (range) were compared using the Mann–Whitney *U* test. The *χ* ^2^ test or Fisher’s exact test was used to calculate statistical differences of categorical variables. All variables related to the MVI and its M2 classification in the univariate analysis were regarded as candidates for multivariate logistic analysis. The least absolute shrinkage and selection operator (LASSO) regression model was used to reduce data dimensionality and select the most significant elements with non-zero coefficients (24). The integrated discrimination improvement (IDI) is the difference in the discrimination slopes for a prediction model with and without one variable, which indicates whether the discrimination slope of a model will improve if one important parameter is added. The net reclassification improvement (NRI) is an index that attempts to quantify how well a new model correctly reclassifies subjects. So IDI and NRI can be used for the comparison between an original model and a new model (the original model plus one additional component) (25).

The final predictors correlated with MVI and its M2 classification were determined by LASSO regression, logistic regression, and IDI and NRI analyses and used to establish the corresponding nomograms. The nomogram can proportionally convert each regression coefficient in the logistic regression to a scale of 0 to 100 points (26). The points of each independent variable were summed, and the predicted probabilities were derived from the total points. The predictive performance and accuracy of the nomograms were evaluated by AUC and calibration curve, respectively. Decision curve analysis (DCA) was performed by calculating the net benefits at different points of threshold probabilities to evaluate the clinical utility of the nomograms. In all analyses, *P <*0.05 was considered to indicate statistical significance. All analyses were performed using SPSS version 22.0 (SPSS Inc, Chicago, IL, USA) and R version 4.0.3.

## Results

### Clinicopathological Characteristics

A total of 111 patients with HCC were retrospectively enrolled in this study. The median age was 57 years (range 37–80), 97 (87.4%) patients were male, 14 (12.6%) patients were female, 40 (36.0%) patients had symptoms at diagnosis, and the median tumor size (longest tumor diameter) was 7 cm (range 1.5–22). Based on the eighth TNM staging system recommended by the AJCC, among 111 HCC patients, 8 cases (7.2%) were classified as stage I, 38 cases (34.2%) were classified as stage II, 60 cases (54.1%) were classified as stage III, and 5 cases (4.5%) were classified as stage IV. MVI was found in 72 of 111 (64.86%) patients, whereas M2 grade was presented in 47 of 72 (65.28%) MVI-positive patients. The detailed clinicopathological characteristics are listed in **Tables 1** and **2**.

**Table 1:** Clinical characteristics of HCC patients and their correlations with MVI status.

Variables	Total (*n* = 111)	MVI negative (*n* = 39)	MVI positive (*n* = 72)	*P*
Age (years), median (range)	57 (37–80)	56 (37–70)	58 (37–80)	0.814
Sex, *n* (%)				0.961
Male	97 (87.4)	34 (87.2)	63 (87.5)	
Female	14 (12.6)	5 (12.8)	9 (12.5)	
Symptoms at diagnosis				0.417
No	71 (64.0)	27 (69.2)	44 (61.1)	
Yes	40 (36.0)	12 (30.8)	28 (38.9)	
Edmondson–Steiner classification, n (%)				**<0.001**
I–II	62 (55.9)	31 (79.5)	31 (43.1)	
III–IV	49 (44.1)	8 (20.5)	41 (56.9)	
Clinical TNM stage, *n* (%)				**<0.001**
I	8 (7.2)	8 (20.5)	0 (0)	
II	38 (34.2)	24 (61.5)	14 (19.4)	
III	60 (54.1)	6 (15.4)	54 (75.0)	
IV	5 (4.5)	1 (2.6)	4 (5.6)	
Cirrhosis, *n* (%)				0.254
No	28 (25.2)	7 (17.9)	21 (29.2)	
Yes	83 (74.8)	32 (82.1)	51 (70.8)	
Tumor number, *n* (%)				**<0.001**
Solitary	61 (55.0)	31 (79.5)	30 (41.7)	
Multiple	50 (45.0)	8 (20.5)	42 (58.3)	
Tumor size (cm), median (range)	7 (1.5–22)	4 (2–14)	10 (1.5–22)	**<0.001**
Tumor capsule, n (%)				**<0.001**
Absent	55 (49.5)	5 (12.8)	50 (69.5)	
Incomplete	23 (20.7)	7 (17.9)	16 (22.2)	
Complete	33 (29.7)	27 (69.2)	6 (8.3)	
Tumor location, *n* (%)				0.699
Right lobe of liver	77 (69.4)	26 (66.7)	51 (70.8)	
Left lobe of liver	26 (23.4)	9 (23.1)	17 (23.6)	
Both lobe of liver	5 (4.5)	2 (5.1)	3 (4.2)	
Caudate lobe	3 (2.7)	2 (5.1)	1 (1.4)	
Tumor margin, *n* (%)				**<0.001**
Not smooth	52 (46.8)	6 (15.4)	46 (63.9)	
Smooth	59 (53.2)	33 (84.6)	26 (36.1)	
Satellite nodule, *n* (%)				**0.003**
Absent	61 (55.0)	29 (74.4)	32 (44.4)	
Present	50 (45.0)	10 (25.6)	40 (55.6)	
HBsAg, n (%)				0.834
Negative	36 (32.4)	12 (30.8)	24 (33.3)	
Positive	75 (67.6)	27 (69.2)	48 (66.7)	
AFP (ng/ml)				**<0.001**
<20	29 (26.1)	19 (48.7)	10 (13.9)	
20–400	30 (27.0)	11 (28.2)	19 (26.4)	
>400	52 (46.8)	9 (23.1)	43 (59.7)	
CEA (ng/ml), median (range)	2.62 (0.2–29.69)	2.9 (0.24–27.56)	2.21 (0.2–29.69)	0.369
ALT (U/L), median (range)	40 (3–209)	41 (8–209)	36.5 (3–108)	0.509
AST (U/L), median (range)	36 (3–383)	33 (4–383)	36.5 (3–149)	0.965
ALB (g/L), median (range)	39 (21–52)	38 (29–51)	39.5 (21–52)	0.260
PT (s), median (range)	11.9 (9.7–22.6)	12 (10.07–22.6)	11.825 (9.7–18.2)	0.413

MVI, microvascular invasion; TNM, tumor-node-metastasis, according to the eighth edition of the AJCC (American Joint Committee on Cancer) cancer staging manual; HBsAg, hepatitis B surface antigen; AFP, alpha fetoprotein; CEA, carcinoembryonic antigen; ALT, alanine aminotransferase; AST, aspartate aminotransferase; ALB, albumin; PT, prothrombin time. P: categorical variables—χ^2^ test or Fisher’s exact test; continuous variables—Mann–Whitney U test. The bold value means statistical significance.

**Table 2:** Clinical characteristics comparison in HCC patients with different degrees of MVI.

Variables	M1 (*n* = 25)	M2 (*n* = 47)	*P*
Age (years), median (range)	60 (37–80)	56 (39–79)	0.705
Sex, *n* (%)			0.710
Male	21 (84.0)	42 (89.4)	
Female	4 (16.0)	5 (10.6)	
Symptoms at diagnosis			0.452
No	17 (68.0)	27 (57.4)	
Yes	8 (32.0)	20 (42.6)	
Edmondson–Steiner classification, n (%)			0.620
I–II	12 (48.0)	19 (40.4)	
III–IV	13 (52.0)	28 (59.6)	
Clinical TNM stage, *n* (%)			**0.017**
I	0 (0)	0 (0)	
II	9 (36.0)	5 (10.6)	
III	16 (64.0)	38 (80.9)	
IV	0 (0)	4 (8.5)	
Cirrhosis, *n* (%)			0.280
No	5 (20.0)	16 (34.0)	
Yes	20 (80.0)	31 (66.0)	
Tumor number, *n* (%)			**0.026**
Solitary	15 (60.0)	15 (31.9)	
Multiple	10 (40.0)	32 (68.1)	
Tumor size (cm), median (range)	11 (2–20)	10 (1.5–22)	0.709
Tumor capsule, n (%)			**<0.001**
Absent	10 (40.0)	40 (85.1)	
Incomplete	10 (40.0)	6 (12.8)	
Complete	5 (20.0)	1 (2.1)	
Tumor location, *n* (%)			0.909
Right lobe of liver	18 (72.0)	33 (70.2)	
Left lobe of liver	6 (24.0)	11 (23.4)	
Both lobe of liver	1 (4.0)	2 (4.3)	
Caudate lobe	0 (0)	1 (2.1)	
Tumor margin, *n* (%)			**0.001**
Not smooth	9 (36.0)	37 (78.7)	
Smooth	16 (64.0)	10 (21.3)	
Satellite nodule, *n* (%)			0.213
Absent	14 (56.0)	18 (38.3)	
Present	11 (44.0)	29 (61.7)	
HBsAg, n (%)			0.861
Negative	8 (32.0)	16 (34.0)	
Positive	17 (68.0)	31 (66.0)	
AFP (ng/ml)			0.194
<20	6 (24.0)	4 (8.5)	
20–400	6 (24.0)	13 (27.7)	
>400	13 (52.0)	30 (63.8)	
CEA (ng/ml), median (range)	2.48 (1.04–29.69)	2.13 (0.2–28.69)	0.538
ALT (U/L), median (range)	44 (11–99)	35 (3–108)	0.456
AST (U/L), median (range)	37 (19–114)	36 (3–149)	0.239
ALB (g/L), median (range)	39 (32–51)	40 (21–52)	0.526
PT (s), median (range)	11.85 (10.6–18.2)	11.7 (9.7–15.2)	0.424

M1 and M2 classification based on the three-tiered microvascular invasion grading system. TNM, tumor-node-metastasis, according to the eighth edition of the AJCC (American Joint Committee on Cancer) cancer staging manual; HBsAg, hepatitis B surface antigen; AFP, alpha fetoprotein; CEA, carcinoembryonic antigen; ALT, alanine aminotransferase; AST, aspartate aminotransferase; ALB, albumin; PT, prothrombin time. P: categorical variables—χ^2^ test or Fisher’s exact test; continuous variables—Mann–Whitney U test. The bold value means statistical significance.

### Independent Significant Factors for the Presence of MVI and Its M2 Grade

In comparison of the clinicopathological characteristics between the MVI-positive and MVI-negative groups, eight variables, namely, clinical TNM stage, α-fetoprotein (AFP), Edmondson–Steiner classification, tumor size, tumor number, tumor capsule, tumor margin, and satellite nodule, were significantly associated with the MVI according to the univariate analysis (**Table 1**). Nevertheless, among MVI-positive cases, only clinical TNM stage, tumor number, tumor capsule, and tumor margin showed statistical correlation with M2 grade (**Table 2**). Furthermore, clinical TNM stage, Edmondson–Steiner classification, tumor size, tumor capsule, tumor margin, and AFP were found to be independent risk factors of MVI by multivariate analysis; interestingly, when MVI was present, three variables of tumor number, tumor capsule, and tumor margin were highly associated with M2 grade from the result of multivariate analysis (**Table 3**).

**Table 3:** Risk factors for MVI and its M2 grade identified by logistic multivariate analysis.

Factors	MVI presence			M2 degree of MVI presence		
	OR	95% CI	*P*	OR	95% CI	*P*
Edmondson–Steiner classification						
I–II	1			1		
III–IV	7.333	1.797–29.922	**0.005**	1.849	0.578–5.909	0.300
Clinical TNM stage						
I–II	1			1		
III–IV	6.031	1.577–23.061	**0.009**	3.906	0.986–15.473	0.052
Tumor number						
Solitary	1			1		
Multiple	3.885	0.817–18.460	0.088	3.200	1.168–8.770	**0.024**
Tumor size (cm)						
<5	1			1		
≥5	5.129	1.081–24.349	**0.040**	2.117	0.413–10.844	0.368
Tumor capsule						
Present	1			1		
Absent	6.174	1.775–21.475	**0.004**	7.772	2.411–25.052	**0.001**
Tumor margin						
Smooth	1			1		
Not smooth	4.999	1.620–15.430	**0.005**	6.578	2.246–19.266	**0.001**
Satellite nodule						
Absent	1			1		
Present	1.155	0.232–5.756	0.860	2.601	0.853–7.932	0.093
AFP (ng/ml)						
<20	1			1		
20–400	4.046	1.129–14.497	**0.032**	4.373	0.771–24.806	0.096
>400	9.322	2.586–33.613	**0.001**	4.089	0.823–20.319	0.085

MVI, microvascular invasion; M2 classification based on the three-tiered MVI grading system. TNM, tumor-node-metastasis, according to the eighth edition of the AJCC (American Joint Committee on Cancer) cancer staging manual; AFP, alpha fetoprotein. The bold value means statistical significance.

### Identification of Predictive Factors by LASSO Regression

**Figure 2:**
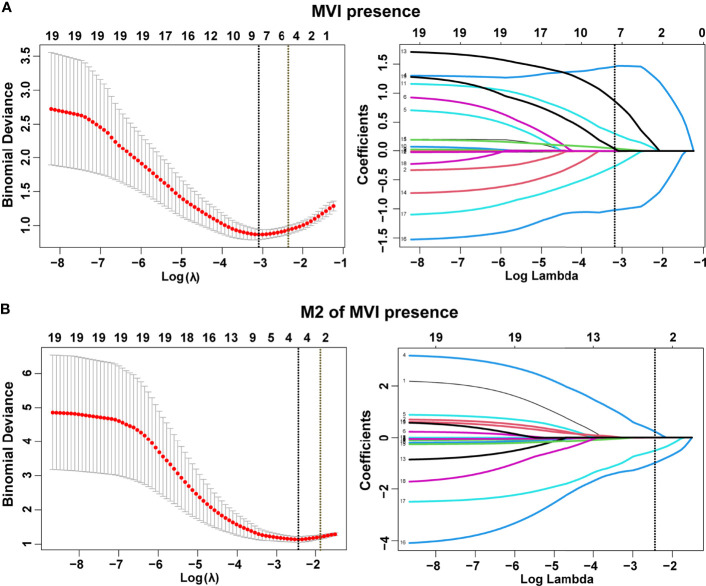
Selection of demographic and clinical features using the least absolute shrinkage and selection operator (LASSO) regression model. Selection of tuning parameter (*λ*) in the LASSO model by three-fold cross-validation based on minimum criteria for MVI **(A)** and its M2 grade **(B)**. Dotted vertical lines were drawn at the optimal values using the minimum criteria and the 1 standard error of the minimum criteria (1-SE criteria). All features with non-zero coefficients are indicated on the right of **(A, B)**.

In total, 19 variables were analyzed by LASSO regression and 8 candidate factors were determined to be associated with MVI (**Figure 2A**). These factors were clinical TNM stage, alanine aminotransferase (ALT), AFP, Edmondson–Steiner classification, tumor size, tumor capsule, tumor margin, and tumor number. Among the patients with MVI presence, clinical TNM stage, tumor capsule, tumor margin, and aspartate transaminase (AST) were selected and identified as risk factors of M2 grade by using LASSO regression analysis (**Figure 2B**). The coefficients of selected parameters associated with MVI and its M2 grade are shown in **Table S1**.

### Confirmation of the Best Prediction Model for MVI and M2 Grade

The base model (model 1) was then created by incorporating six variables (Edmondson–Steiner classification, clinical TNM stage, tumor size, tumor capsule, tumor margin, and AFP) determined to be associated with MVI both in logistic and LASSO analyses. By severally adding ALT and tumor number to model 1, we constructed two new models named model 2 and model 3. Taking model 1 as the reference, model 2 did not exhibit superiority for predicting MVI. Adding tumor number to model 1 did not appreciably change the AUC and IDI, but led to a significant improvement in the continuous NRI (cNRI) (**Table 4**), which indicated that model 2 was superior to model 1 in MVI prediction. Moreover, among HCC patients with MVI presence, tumor capsule and tumor margin were both confirmed by LASSO and logistic regression to be related with M2 grade. So the second base model (model A) was established by incorporating tumor capsule and tumor margin. Subsequently, we developed model B, model C, and model D by respectively adding clinical TNM stage, AST, and tumor number to the base model A and found that model B and model D are better than model A for predicting M2 grade in the presence of MVI (model B vs. model A, cNRI = 0.507, *p* = 0.017; model D vs. model A, cNRI = 0.562, *p* = 0.019), whereas model C did not show any superiority in M2 prediction (**Table 4**), suggesting that clinical TNM stage and tumor number can be definitely considered as the risk factors of M2 grade when MVI is present.

**Table 4:** Comparison of different prediction models for estimating the risk of MVI and its M2 grade.

Model	AUC (95% CI)	*P*	IDI (95% CI)	*P*	cNRI (95% CI)	*P*
**MVI positive**						
Model 1 (base model)	0.921 (0.868–0.975)	Ref		Ref		Ref
Model 2	0.925 (0.877–0.975)	0.562	−0.001 (−0.013 to 0.012)	0.984	0.256 (−0.125 to 0.638)	0.187
Model 3	0.926 (0.877–0.974)	0.569	0.003 (−0.012 to 0.018)	0.683	0.756 (0.416 to 1.097)	**<0.001**
**M2 grade of MVI**						
Model A (base model)	0.764 (0.649–0.879)	Ref		Ref		
Model B	0.799 (0.691–0.908)	0.175	0.024 (−0.021 to 0.069)	0.291	0.507 (0.092 to 0.923)	**0.017**
Model C	0.778 (0.660–0.896)	0.533	0.004 (−0.002 to 0.009)	0.203	0.238 (−0.225 to 0.701)	0.313
Model D	0.773 (0.652–0.893)	0.684	0.008 (−0.012 to 0.027)	0.454	0.562 (0.094 to 1.029)	**0.019**

Model 1 = Edmondson–Steiner classification + clinical TNM stage + tumor size + tumor capsule + tumor margin + AFP; model 2 = model 1 + ALT; model 3 = model 1 + tumor number; model A = tumor capsule + tumor margin; model B = model A+ clinical TNM stage; model C = model A + AST; model D = model A + tumor number.

AUC, area under curve; IDI, integrated discrimination improvement; cNRI, continuous net reclassification improvement. The bold value means statistical significance.

### Development and Validation of Nomograms for Predicting MVI and Its M2 Grade

**Figure 3:**
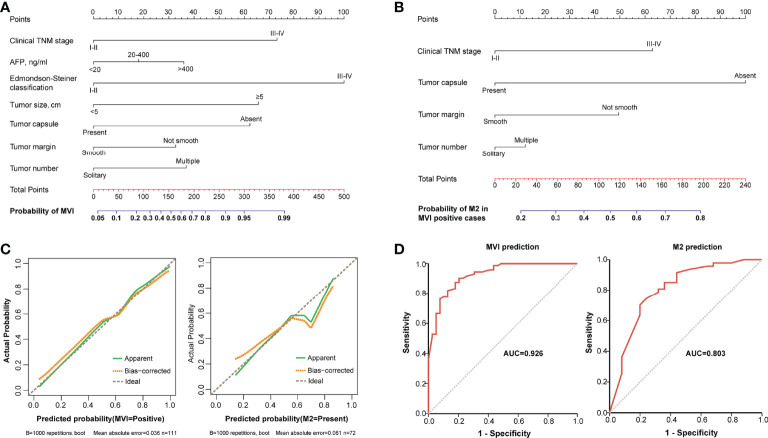
The nomograms and their calibration and discrimination. **(A)** The MVI nomogram was built by incorporating clinical TNM stage, AFP, Edmondson–Steiner classification, tumor size, tumor capsule, tumor margin, and tumor number. **(B)** Among the MVI-positive cases, clinical TNM stage, tumor capsule, tumor margin, and tumor number were used to establish another nomogram for predicting M2 grade. Locate the patient’s characteristic on a variable row and draw a vertical line straight up to the points’ row (top) to assign a point value for the variable. Adding up the total number of points and drop a vertical line from the total points’ row to obtain the probability of predictive outcomes. **(C)** The calibration curves of the two nomograms based on internal validation with a bootstrap resampling frequency of 1,000. **(D)** The ROC curves with AUCs of 0.926 and 0.803 to demonstrate the discriminatory ability of the two nomograms.

A nomogram incorporating Edmondson–Steiner classification, clinical TNM stage, tumor size, tumor capsule, tumor margin, AFP, and tumor number was constructed for MVI prediction (**Figure 3A**). In the presence of MVI, a second nomogram for predicting M2 grade was developed by using four variables, namely, clinical TNM stage, tumor capsule, tumor margin, and tumor number (**Figure 3B**). Calibration curves of the two nomograms demonstrated good consistency between the predicted and observed results regarding the MVI status and its M2 classification (**Figure 3C**). The AUC of the nomogram predicting MVI was 0.926, and the AUC of the nomogram for M2 grade prediction in MVI-positive cases was 0.803 (**Figure 3D**).

### Clinical Use of the Nomograms for MVI and Its M2 Grade Prediction

**Figure 4:**
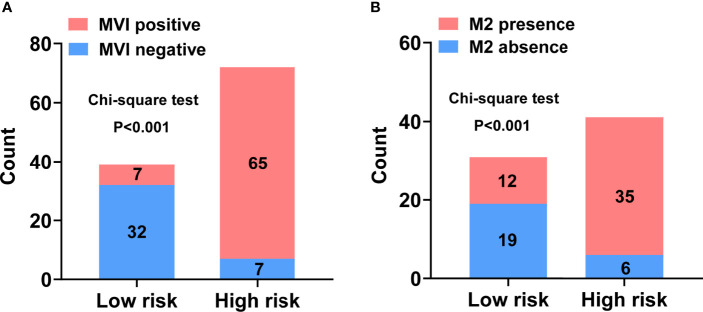
Discriminatory power of the nomograms for MVI and its M2 grade with bar charts. Risk classification of the predictive nomograms conducted by the maximum Youden index, and the performance in distinguishing the MVI **(A)** and its M2 grade **(B)**. *P*-values were calculated by the chi-square test.

**Figure 5:**
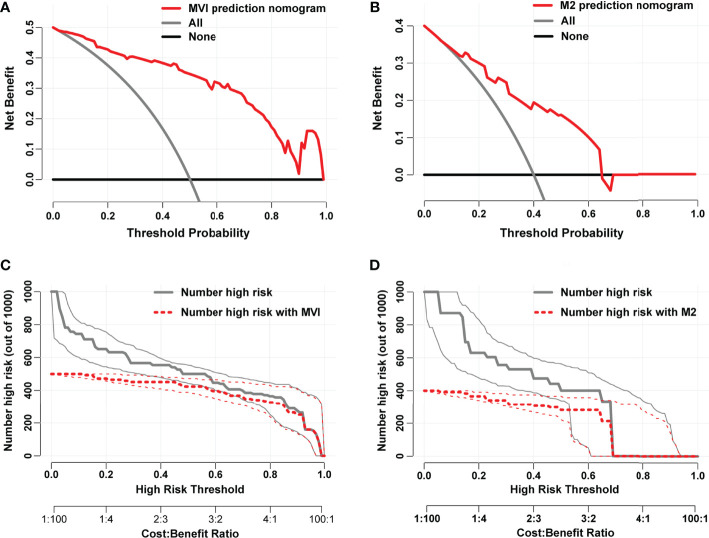
Decision curves of the nomograms for predicting presence of MVI **(A)** and its M2 grade **(B)**. The horizontal solid black line represents the hypothesis that no patients experienced the presence of MVI or its M2 grade, and the solid gray line represents the hypothesis that all patients met the endpoint. Clinical impact curves of the nomograms for MVI and its M2 grade prediction, respectively, were plotted in **(C, D)**. At different threshold probabilities within a given population, the number of high-risk patients and the number of high-risk patients with the outcome were shown.

Each variable displayed in the two nomograms was assigned a risk score. The detailed scores of these variables are presented in **Table S2**. The final total scores that ranged from 0 to 407 (MVI nomogram) and 0 to 225 (M2 nomogram) were obtained by summing the scores of each variable. The optimal cutoff values of the total scores were confirmed by the maximum Youden index in ROC curve analysis (**Tables S3** and **S4**). Based on the cutoff scores of 172 from the MVI nomogram and 163 from the M2 nomogram, HCC patients were being divided into low- and high-risk groups. The high-risk groups had a significantly greater probability of having MVI and were classified as M2 grade (**Figure 4**). Then DCA results revealed that using the two nomograms to predict MVI and its M2 grade for almost all threshold probabilities at different points added more net benefit than the treat-all or treat-none strategies (**Figures 5A, B**), suggesting good clinical utility of the two nomograms. For the purpose of understanding their significance more intuitively, clinical impact curves of the nomograms for prediction in MVI and its M2 grade were plotted (**Figures 5C, D**), and the distance between the curve of the high-risk predicted number (the gray curve) and the curve of the high-risk actual number (the red curve) was very close in almost all high-risk threshold points, indicating that the two models had remarkable predictive power.

## Discussion

The long-term prognosis of HCC patients at early- to intermediate-stage after curative therapies is still poor, mainly due to the high recurrence rate after primary resection (7). Being considered as an important marker of HCC aggressive behavior, MVI could greatly affect intrahepatic metastasis of tumor cells *via* the portal circulation (27) and lead to tumor recurrence after curative surgery (28). Among MVI-positive cases, the M2 grade is an obvious indicator of HCC poor prognosis. The tumor microenvironment of HCC with MVI-M2 grade provides a favorable condition for tumor rapid growth and aggressive invasion, resulting in a true R0 surgical resection which is difficult to achieve (18). MVI and its M2 classification based on MVI-TTG only can be diagnosed by histopathological examination after surgical resection (17). Hence, it is important to find the significant risk factors of MVI and its M2 grade and develop prediction models by using these factors, which could provide optimal management decision. In the present study, approximately 64.86% of patients (72/111) with HCC harbored MVI, and among these MVI-positive cases, 47 patients (65.28%) were classified as M2 grade. Our analysis also suggested that later clinical tumor stage, higher AFP, more advanced Edmondson–Steiner classification, larger tumor size, tumor capsule absence, non-smooth tumor margin, and multiple tumor number were significantly associated with MVI, and patients with MVI-M2 among these MVI-positive cases were more likely to have later clinical tumor stage, absent tumor capsule, non-smooth tumor margin, and multiple tumor number.

Almost all studies indicated that tumor size was associated with MVI. However, the correlation between tumor size classification and MVI remained controversial. A study from an international multicenter database showed that the incidence of MVI increased with the tumor size of resected HCC (tumor size, MVI incidence: ≤3 cm, 25%; 3.1–5 cm, 40%; 5.1–6.5 cm, 55%; >6.5 cm, 63%) (29). Kim et al. (30) and Siegel et al. (31) respectively reported that tumor size more than 2 or 3 cm was a risk factor of MVI. In our study, we found that tumor size was also correlated with MVI; especially HCCs more than 5 cm increased the probability of MVI formation. Interestingly, in MVI-positive patients, there is no significant difference in tumor size between M1 and M2 grades. It is speculated that tumor size may only play a role in tumor cells if they can invade the microvessels. Once MVI is present, the tumor size perhaps has little to do with the site number of MVI. According to histological examination, MVI-positive tumors have a strong aggressive tendency to invade the tumor encapsulation, making the tumor margin irregular (32). Among MVI-positive patients, 67% HCCs were found irregular or had non-smooth margin (33). Consistently, in this study, absent tumor capsule and non-smooth tumor margin were demonstrated to be the independent risk factors of MVI. Besides, we also found that tumor capsule and tumor margin have a significant difference between M1 and M2 grades in MVI-positive cases.

What is more, several studies have identified that multiple tumors and elevated AFP levels are associated with an increased probability of vascular invasion in HCCs (9, 34, 35). Similarly, our study demonstrated that AFP was also significantly associated with MVI presence, but the tumor number did not seem to be an independent risk factor of MVI. The *P*-value (0.088) of tumor number in multivariate analysis for MVI risk factor estimation was close to 0.05, which might show statistical significance if this study had a much larger sample size. In contrast, high serum AFP level did not appear to correlate with MVI-M2 grade, while MVI-positive patients with multiple tumors were more likely to be M2 grade according to our multivariate logistic analysis, meaning that the serum AFP level does not affect the number of MVI sites but cases with multiple tumors have more MVI sites. Clinical TNM stage is an important reference for evaluating the prognosis of HCC patients and is a comprehensive variable that integrates tumor size, number of tumor lesions, lymph node metastasis, and distant metastasis, and generally assessed by radiological imaging before surgery. The Edmondson–Steiner classification represents the degree of HCC differentiation. The preoperative true diagnosis of HCC is usually by liver biopsy. When HCC diagnosis was confirmed, information of the HCC differentiation also can be obtained simultaneously. In multivariate logistic analysis, we found that clinical TNM stage and Edmondson–Steiner classification were significantly related to MVI yet not the independent predictors of MVI-M2 grade. From the result of multivariate logistic analysis for M2 risk factors, clinical TNM stage *P*-value (0.052) was extremely close to 0.05. Meanwhile, given the remarkable impact of clinical TNM stage on the prognosis of HCC patients, we speculated that this variable may also be a predictor of MVI-M2 grade.

Subsequently, we found that clinical TNM stage, AFP, Edmondson–Steiner classification, tumor size, tumor capsule, tumor margin, tumor number, and ALT were risk factors for MVI formation based on the LASSO regression analysis. Also, in patients with MVI presence, clinical TNM stage, AST, tumor capsule, and tumor margin were related to M2 classification. However, in the logistic regression results, ALT and tumor number were not independent risk factors for MVI, and clinical stage and AST were not associated with M2 grade when MVI was present. In addition, according to the LASSO regression results, there was no correlation between tumor number and M2 grade among MVI-positive patients. Therefore, in order to further explore whether ALT and tumor number are independent risk factors for the formation of MVI, we established model 2 and model 3 by respectively adding ALT and tumor number to model 1 (base model incorporating clinical TNM stage, AFP, Edmondson–Steiner classification, tumor size, tumor capsule, and tumor margin). The cNRI analysis revealed a remarkable MVI prediction improvement in model 3, whereas no significant MVI prediction improvement was observed in model 2 compared with model 1, which means that tumor number can be considered as an independent predictive factor of MVI presence. Additionally, to further clarify whether clinical stage, tumor number, and AST correlate with M2 classification in the presence of MVI, we constructed three new models named model B, model C, and model D by adding clinical tumor stage, AST, and tumor number, respectively, to model A (base model including tumor capsule and tumor margin). Taking model A as reference, the cNRI analysis showed that model B and model D significantly improved the M2 prediction, but model C did not have improvement of predictive ability for M2 in MVI-positive cases, indicating that clinical tumor stage and tumor number are the true predictors of M2 grade.

The nomogram has been recognized as a user-friendly and practical prediction tool with high accuracy and good discriminative power and is widely used in the evaluation of prognosis or an outcome event (36, 37). Hence, a nomogram was developed for MVI prediction by incorporating Edmondson–Steiner classification, clinical TNM stage, tumor margin, AFP level, tumor size, tumor capsule, and tumor number, and a nomogram including clinical TNM stage, tumor capsule, tumor margin, and tumor number was also built for predicting M2 classification in the presence of MVI. Both nomograms demonstrated good consistency between the predicted probabilities and the actual observations according to the optimal calibration curves. Furthermore, satisfactory diagnostic performance was found in these two nomograms with AUCs of 0.926 (AUC_MVI_) and 0.803 (AUC_M2_). Then the cutoff values of total points were determined as 172 in the MVI nomogram and 163 in the M2 nomogram according to the maximum Youden index from ROC analysis. Patients with a total score of >172 were a high-risk subgroup of MVI and those with a score of >163 were considered as high-risk of M2 grade when MVI was present, which could guide us to make the best treatment decision. Moreover, we also opted to conduct a DCA to determine the clinical utility of our nomogram. DCA is a novel method to evaluate the clinical benefits of diagnostic tests and prediction models (38). Here, great net benefit of the established nomograms with the risk threshold more than 0.2 was shown in DCA, indicating good clinical utility of the two nomograms. In addition, excellent predictive power of the two nomograms was further determined by plotting the clinical impact curves.

Although most previous studies generally split the dataset randomly into two groups of training set and validation set, this was not adopted in our study due to limitation of sample size. Besides, this approach did not fully utilize all available data to develop the prediction model, resulting in statistical inefficiency or even waste (39). HCC patients of our study both had positive and negative HBsAg, and the established nomogram showed satisfactory discriminative performance regardless of HBV infection, indicating that our prediction model might comparably be suitable for HCC caused by viral hepatitis and non-viral hepatitis. It is a pity that this study still had several limitations. Firstly, this was a retrospective study with a small sample size and had an inevitable case selection bias. So a large sample size prospective study with balanced populations is required to further confirm the reliability of our nomograms in the future. Secondly, our study was only conducted at a single institute and did not have any validation. It is necessary to validate our results by using data from multiple centers. Finally, the nomograms were established just based on the limited clinical data; thus, specific genetic markers need to be identified and incorporated into nomograms to further advance the prediction accuracy of the nomograms.

In conclusion, the Edmondson–Steiner classification, clinical TNM stage, tumor margin, AFP level, tumor size, tumor capsule, and tumor number were identified as significant predictive factors for MVI in HCC patients, and clinical TNM stage, tumor capsule, tumor margin, and tumor number were confirmed as independent predictors of M2 grade among MVI-positive cases. Then two wieldy nomograms were developed by incorporating these variables above, making individualized prediction of MVI and its M2 grade more objective and accurate. Judging from the two nomogram scoring systems, more aggressive treatment may be recommended to reduce potential future recurrence if patients are considered as high risk of MVI and perhaps classified into M2 grade. Last but not least, our nomograms can improve individualized therapy design and facilitate monitoring plan selection, which may lead to effective and curative treatment initiation for HCC patients.

## Data Availability Statement

The raw data supporting the conclusions of this article will be made available by the authors, without undue reservation.

## Ethics Statement

The studies involving human participants were reviewed and approved by the Ethics Committee of Zhejiang Cancer Hospital. Written informed consent for participation was not required for this study in accordance with the national legislation and the institutional requirements.

## Author Contributions

SW and SC conceived the idea and designed the study. RR and JY collected the data. CW and YG analyzed the data. SC, CW, and YG drafted the manuscript. All authors contributed to the article and approved the submitted version.

## Funding

This study was funded by the Medical Health Science and Technology Project of Zhejiang Province(No.2022KY619).

## Conflict of Interest

The authors declare that the research was conducted in the absence of any commercial or financial relationships that could be construed as a potential conflict of interest.

## Publisher’s Note

All claims expressed in this article are solely those of the authors and do not necessarily represent those of their affiliated organizations, or those of the publisher, the editors and the reviewers. Any product that may be evaluated in this article, or claim that may be made by its manufacturer, is not guaranteed or endorsed by the publisher.
