# Corrigendum: Prediction of Microvascular Invasion and Its M2 Classification in Hepatocellular Carcinoma Based on Nomogram Analyses

**DOI:** 10.3389/fonc.2022.888008

**Published:** 2022-04-05

**Authors:** Shengsen Chen, Chao Wang, Yuwei Gu, Rongwei Ruan, Jiangping Yu, Shi Wang

**Affiliations:** ^1^ Department of Endoscopy, Cancer Hospital of the University of Chinese Academy of Sciences(Zhejiang Cancer Hospital), Institute of Cancer and Basic Medicine (IBMC), Chinese Academy of Sciences, Hangzhou, China; ^2^ Department of Emergency, Huashan Hospital affiliated to Fudan University, Shanghai, China; ^3^ Department of Rehabilitation Medicine, Huashan Hospital affiliated to Fudan University, Shanghai, China

**Keywords:** hepatocellular carcinoma, microvascular invasion, M2 classification, prediction model, nomogram

There is an error in the Funding statement. The correct number for the Medical Health Science and Technology Project of Zhejiang Province is 2022KY619. There is also an error in the original article. This mistake is in the abstract methods part, in the sentence “A total of 111 patients who underwent radical resection of hepatocellular carcinoma (HCC) from January 2015 to September 2020 were retrospectively collected”. Here the time is wrong, it should be from January 2017 to December 2019 instead.

A correction has been made to **Abstract** section, **Methods** sub-section, first paragraph:

“A total of 111 patients who underwent radical resection of hepatocellular carcinoma (HCC) from January 2017 to December 2019 were retrospectively collected. We utilized logistic regression and least absolute shrinkage and selection operator (LASSO) regression to identify the independent predictive factors of MVI and its M2 classification. Integrated discrimination improvement (IDI) and net reclassification improvement (NRI) were calculated to select the potential predictive factors from the results of LASSO and logistic regression. Nomograms for predicting MVI and its M2 grade were then developed by incorporating these factors. Area under the curve (AUC), calibration curve, and decision curve analysis (DCA) were respectively used to evaluate the efficacy, accuracy, and clinical utility of the nomograms.”

The authors apologize for this error and state that this does not change the scientific conclusions of the article in any way. The original article has been updated.

## Publisher’s Note

All claims expressed in this article are solely those of the authors and do not necessarily represent those of their affiliated organizations, or those of the publisher, the editors and the reviewers. Any product that may be evaluated in this article, or claim that may be made by its manufacturer, is not guaranteed or endorsed by the publisher.

