# Supplementary material for: Prediction of Microvascular Invasion and Its M2 Classification in Hepatocellular Carcinoma Based on Nomogram Analyses
Source: Front Oncol. 2022 Jan 14;11:774800. doi: 10.3389/fonc.2021.774800 (PMC8796824; doi:10.3389/fonc.2021.774800)
Supplement: Supplementary file 1 [file Table_1.docx]

| **Table S1.** Coefficients for selected features in the LASSO regression models on MVI and M2 status | | | |
| --- | --- | --- | --- |
| MVI presence λmin= 0.04505879, In(λ)= -3.099787 | | M2 λmin= 0.08754002, In(λ)= -2.435659 | |
| Index | coefficient | Index | coefficient |
| Clinical TNM stage | 1.466889602 | Clinical TNM | 0.227072968 |
| ALT | -0.002871792 | AST | -0.002810743 |
| AFP | 0.274686041 | Tumor capsule | -0.976420357 |
| Edmondson-Steiner classification | 0.810449288 | Tumor margin | -0.501474273 |
| Tumor size | 0.037867674 |  |  |
| Tumor capsule | -1.011975605 |  |  |
| Tumor margin | -0.236814913 |  |  |
| Tumor number | 0.004239073 |  |  |

| **Table S2.** Point assignments and predictive scores for each variable in the nomogram models | | |
| --- | --- | --- |
| Variables | Nomogram score | |
|  | MVI presence | M2 grade of MVI |
| Edmondson-Steiner classification |  |  |
| I-II | 0 | NA |
| III-IV | 100 | NA |
| Clinical TNM stage |  |  |
| Ⅰ-II | 0 | 0 |
| ⅡI-IV | 73 | 63 |
| Tumor number |  |  |
| Solitary | 0 | 0 |
| Multiple | 37 | 12 |
| Tumor size(cm) |  |  |
| <5 | 0 | NA |
| ≥5 | 66 | NA |
| Tumor capsule |  |  |
| Present | 0 | 0 |
| Absent | 62 | 100 |
| Tumor margin |  |  |
| Smooth | 0 | 0 |
| Not smooth | 33 | 50 |
| AFP(ng/ml) |  |  |
| <20 | 0 | NA |
| 20-400 | 18 | NA |
| >400 | 36 | NA |
| NA, not available | | |

| **TableS3.** Identification of the optimal cutoff value of the total nomogram scores for MVI prediction in ROC curve | | | | | |
| --- | --- | --- | --- | --- | --- |
| **Criterion** | **Sensitivity%** | **95% CI** | **Specificity%** | **95% CI** | **Youden index** |
| ≥0 | 100.00 | 95.0 - 100.0 | 0.00 | 0.0 - 9.0 | 0 |
| >0 | 100.00 | 95.0 - 100.0 | 20.51 | 9.3 - 36.5 | 0.2051 |
| >18 | 100.00 | 95.0 - 100.0 | 30.77 | 17.0 - 47.6 | 0.3077 |
| >33 | 100.00 | 95.0 - 100.0 | 33.33 | 19.1 - 50.2 | 0.3333 |
| >36 | 100.00 | 95.0 - 100.0 | 38.46 | 23.4 - 55.4 | 0.3846 |
| >37 | 100.00 | 95.0 - 100.0 | 43.59 | 27.8 - 60.4 | 0.4359 |
| >51 | 100.00 | 95.0 - 100.0 | 46.15 | 30.1 - 62.8 | 0.4615 |
| >55 | 100.00 | 95.0 - 100.0 | 48.72 | 32.4 - 65.2 | 0.4872 |
| >66 | 100.00 | 95.0 - 100.0 | 51.28 | 34.8 - 67.6 | 0.5128 |
| >84 | 98.61 | 92.5 - 100.0 | 56.41 | 39.6 - 72.2 | 0.5502 |
| >99 | 98.61 | 92.5 - 100.0 | 58.97 | 42.1 - 74.4 | 0.5758 |
| >100 | 97.22 | 90.3 - 99.7 | 61.54 | 44.6 - 76.6 | 0.5876 |
| >102 | 94.44 | 86.4 - 98.5 | 61.54 | 44.6 - 76.6 | 0.5598 |
| >103 | 94.44 | 86.4 - 98.5 | 64.10 | 47.2 - 78.8 | 0.5854 |
| >118 | 93.06 | 84.5 - 97.7 | 66.67 | 49.8 - 80.9 | 0.5973 |
| >136 | 91.67 | 82.7 - 96.9 | 69.23 | 52.4 - 83.0 | 0.609 |
| >137 | 91.67 | 82.7 - 96.9 | 71.79 | 55.1 - 85.0 | 0.6346 |
| >139 | 90.28 | 81.0 - 96.0 | 76.92 | 60.7 - 88.9 | 0.672 |
| >157 | 90.28 | 81.0 - 96.0 | 79.49 | 63.5 - 90.7 | 0.6977 |
| **>172** | **90.28** | **81.0 - 96.0** | **82.05** | **66.5 - 92.5** | **0.7233** |
| >175 | 87.50 | 77.6 - 94.1 | 82.05 | 66.5 - 92.5 | 0.6955 |
| >179 | 87.50 | 77.6 - 94.1 | 84.62 | 69.5 - 94.1 | 0.7212 |
| >180 | 84.72 | 74.3 - 92.1 | 84.62 | 69.5 - 94.1 | 0.6934 |
| >197 | 83.33 | 72.7 - 91.1 | 84.62 | 69.5 - 94.1 | 0.6795 |
| >198 | 81.94 | 71.1 - 90.0 | 84.62 | 69.5 - 94.1 | 0.6656 |
| >199 | 80.56 | 69.5 - 88.9 | 84.62 | 69.5 - 94.1 | 0.6518 |
| >202 | 79.17 | 68.0 - 87.8 | 89.74 | 75.8 - 97.1 | 0.6891 |
| >208 | 77.78 | 66.4 - 86.7 | 89.74 | 75.8 - 97.1 | 0.6752 |
| >212 | 76.39 | 64.9 - 85.6 | 92.31 | 79.1 - 98.4 | 0.687 |
| >213 | 73.61 | 61.9 - 83.3 | 92.31 | 79.1 - 98.4 | 0.6592 |
| >227 | 72.22 | 60.4 - 82.1 | 92.31 | 79.1 - 98.4 | 0.6453 |
| >228 | 69.44 | 57.5 - 79.8 | 92.31 | 79.1 - 98.4 | 0.6175 |
| >235 | 68.06 | 56.0 - 78.6 | 92.31 | 79.1 - 98.4 | 0.6037 |
| >245 | 66.67 | 54.6 - 77.3 | 92.31 | 79.1 - 98.4 | 0.5898 |
| >252 | 65.28 | 53.1 - 76.1 | 92.31 | 79.1 - 98.4 | 0.5759 |
| >257 | 63.89 | 51.7 - 74.9 | 92.31 | 79.1 - 98.4 | 0.562 |
| >270 | 61.11 | 48.9 - 72.4 | 92.31 | 79.1 - 98.4 | 0.5342 |
| >271 | 56.94 | 44.7 - 68.6 | 94.87 | 82.7 - 99.4 | 0.5181 |
| >275 | 52.78 | 40.7 - 64.7 | 94.87 | 82.7 - 99.4 | 0.4765 |
| >289 | 50.00 | 38.0 - 62.0 | 94.87 | 82.7 - 99.4 | 0.4487 |
| >297 | 50.00 | 38.0 - 62.0 | 97.44 | 86.5 - 99.9 | 0.4744 |
| >307 | 34.72 | 23.9 - 46.9 | 100.00 | 91.0 - 100.0 | 0.3472 |
| >308 | 33.33 | 22.7 - 45.4 | 100.00 | 91.0 - 100.0 | 0.3333 |
| >312 | 31.94 | 21.4 - 44.0 | 100.00 | 91.0 - 100.0 | 0.3194 |
| >337 | 30.56 | 20.2 - 42.5 | 100.00 | 91.0 - 100.0 | 0.3056 |
| >345 | 29.17 | 19.0 - 41.1 | 100.00 | 91.0 - 100.0 | 0.2917 |
| >356 | 26.39 | 16.7 - 38.1 | 100.00 | 91.0 - 100.0 | 0.2639 |
| >370 | 19.44 | 11.1 - 30.5 | 100.00 | 91.0 - 100.0 | 0.1944 |
| >371 | 16.67 | 8.9 - 27.3 | 100.00 | 91.0 - 100.0 | 0.1667 |
| >389 | 11.11 | 4.9 - 20.7 | 100.00 | 91.0 - 100.0 | 0.1111 |
| >407 | 0.00 | 0.0 - 5.0 | 100.00 | 91.0 - 100.0 | 0 |

| **TableS4.** Identification of the optimal cutoff value of the total nomogram scores for M2 grade prediction in MVI positive cases based on ROC curve | | | | | |
| --- | --- | --- | --- | --- | --- |
| **Criterion** | **Sensitivity%** | **95% CI** | **Specificity%** | **95% CI** | **Youden index** |
| ≥0 | 100.00 | 92.5 - 100.0 | 0.00 | 0.0 - 13.7 | 0 |
| >0 | 97.87 | 88.7 - 99.9 | 20.00 | 6.8 - 40.7 | 0.1787 |
| >62 | 97.87 | 88.7 - 99.9 | 24.00 | 9.4 - 45.1 | 0.2187 |
| >63 | 95.74 | 85.5 - 99.5 | 48.00 | 27.8 - 68.7 | 0.4374 |
| >75 | 91.49 | 79.6 - 97.6 | 56.00 | 34.9 - 75.6 | 0.4749 |
| >100 | 85.11 | 71.7 - 93.8 | 56.00 | 34.9 - 75.6 | 0.4111 |
| >112 | 85.11 | 71.7 - 93.8 | 60.00 | 38.7 - 78.9 | 0.4511 |
| >113 | 85.11 | 71.7 - 93.8 | 64.00 | 42.5 - 82.0 | 0.4911 |
| >125 | 78.72 | 64.3 - 89.3 | 64.00 | 42.5 - 82.0 | 0.4272 |
| >150 | 76.60 | 62.0 - 87.7 | 72.00 | 50.6 - 87.9 | 0.486 |
| **>163** | **74.47** | **59.7 - 86.1** | **76.00** | **54.9 - 90.6** | **0.5047** |
| >175 | 70.21 | 55.1 - 82.7 | 80.00 | 59.3 - 93.2 | 0.5021 |
| >213 | 53.19 | 38.1 - 67.9 | 80.00 | 59.3 - 93.2 | 0.3319 |
| >225 | 0.00 | 0.0 - 7.5 | 100.00 | 86.3 - 100.0 | 0 |
